# Supplementary material for: Double and single stranded detection of 5-methylcytosine and 5-hydroxymethylcytosine with nanopore sequencing
Source: Commun Biol. 2025 Feb 15;8:243. doi: 10.1038/s42003-025-07681-0 (PMC11830040; doi:10.1038/s42003-025-07681-0)
Supplement: Supplementary file 1 — Supplementary Information [file 42003_2025_7681_MOESM1_ESM.docx]

## Supplementary Figures


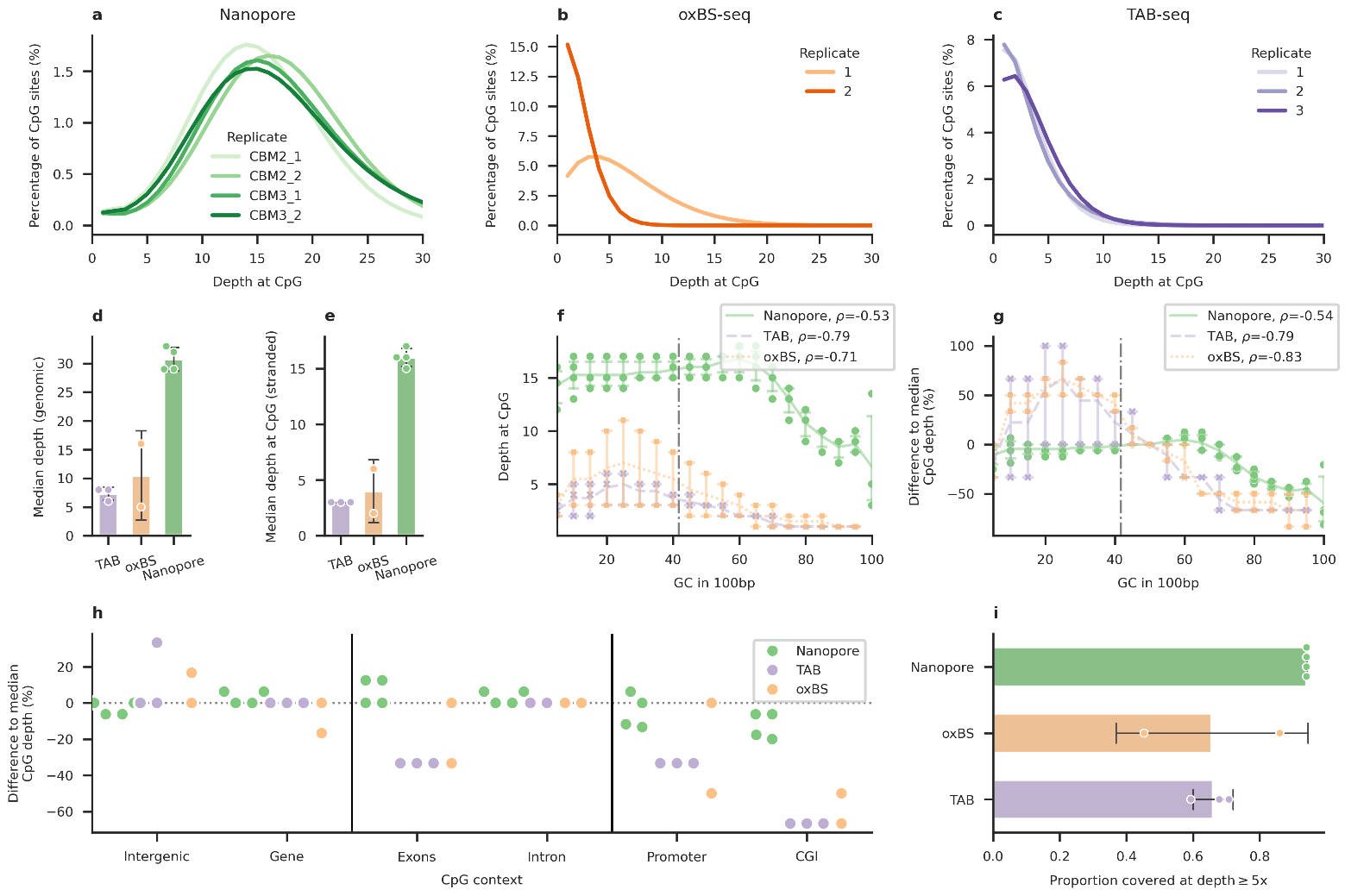


Supplementary Figure 1: Depth and breadth of coverage for whole mouse genome datasets. a-c) Depth of coverage at CpG positions (stranded) for each replicate in the a) nanopore sequence dataset, b) oxBS-seq dataset, or c) TAB-seq dataset. d) Comparison of median genomic sequencing depth depths across techniques, counting reads from either strand. e) Median depth at CpG positions, counting cytosine positions on opposing strands separately. f) Median CpG depth (stranded) as a function of local GC content. CpG sites were grouped into non-overlapping 100bp windows, from which GC percentage is calculated using the mm39 (GRCm39) reference genome. GC percentage is binned in 5-percentile intervals. The depth of matched CpG positions across replicates are plotted separately. Vertical line indicates genomic mean GC percentage of 41.7% in mouse. g) Percentage difference to the median CpG depth of a sequencing replicate within the bins defined in f). h) Percent difference between median CpG depth over different genomic contexts and median CpG depth of a sequencing replicate. i) Proportion of genome covered in at least 5x genomic depth (non-strand specific) by replicate.


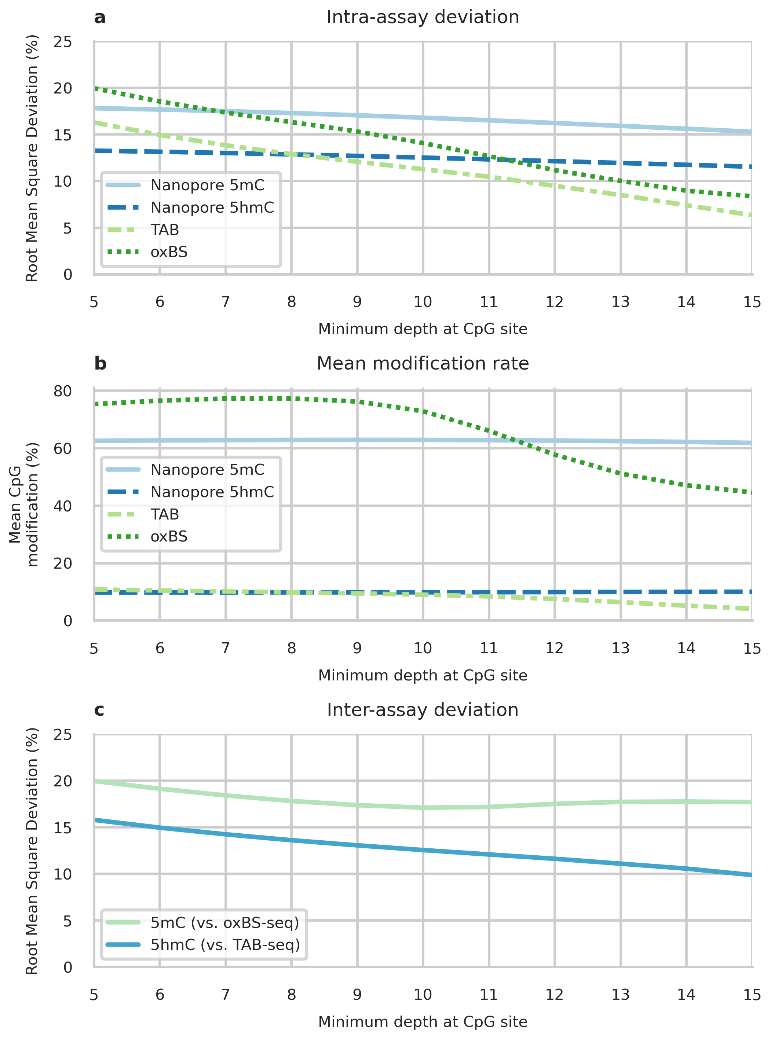


Supplementary Figure 2: Benchmark dataset comparisons with sliding thresholds of minimum depth. Variation is calculated using the Root Mean Square Deviation (RMSD) between all possible dataset pairs, from which a mean is calculated to summarise all permutations. a) Variation in intra-assay variation as a function of depth. b) Change in mean CpG modification percentage over the same sliding depth threshold. c) Variation in inter-assay deviation between the nanopore datasets and respective bisulphite method for 5mC and 5hmC.


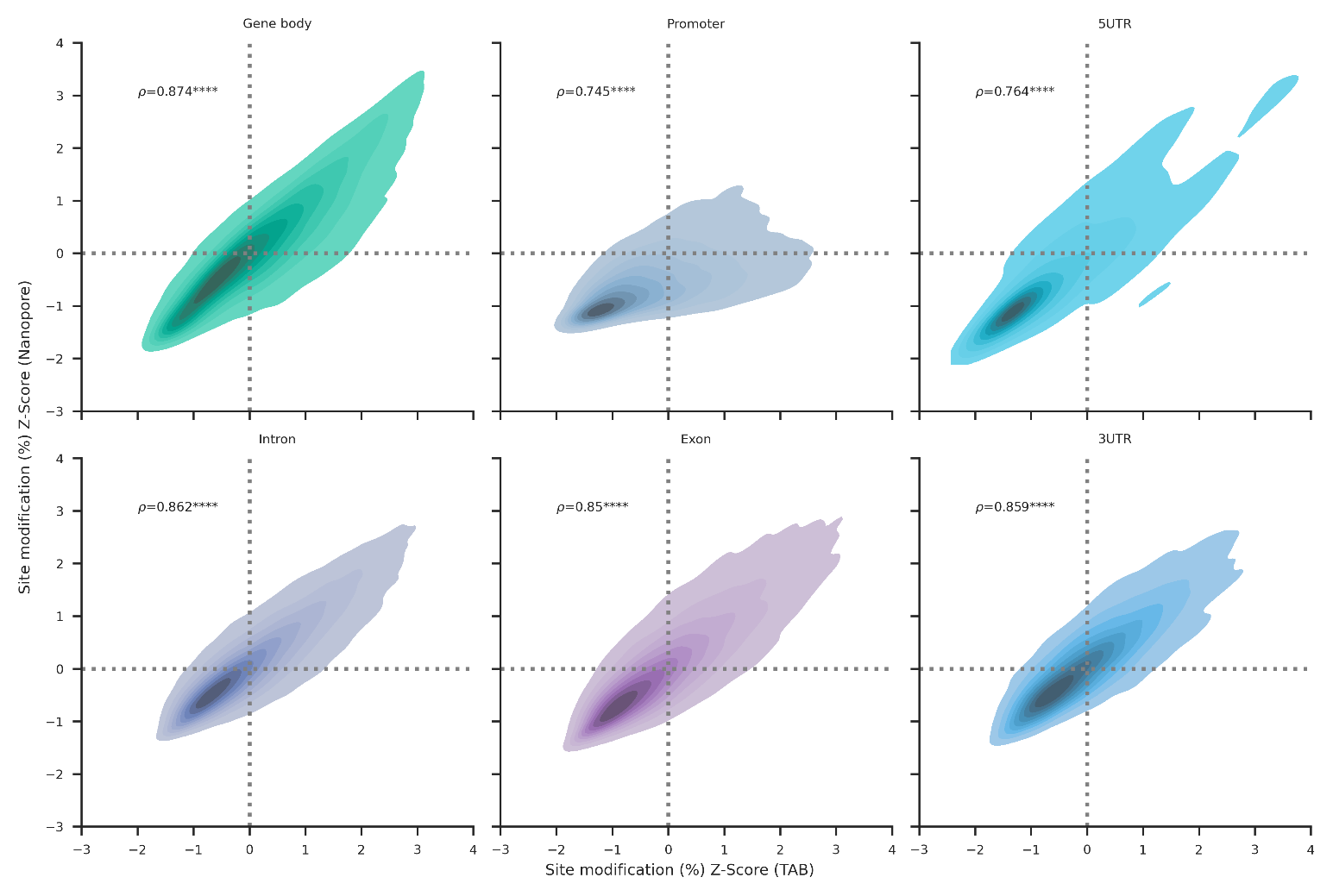


Supplementary Figure 3: 5hmC enrichment in matched genomic features as detected by nanopore sequencing (y) and TAB-seq (x). CpG-context base-calls from both datasets are aggregated across matched genomic features. The proportion of all base-calls within each feature comprised of 5hmC is arsine transformed, with the transformed value used to calculate a Z-score. **** p < 0.0001


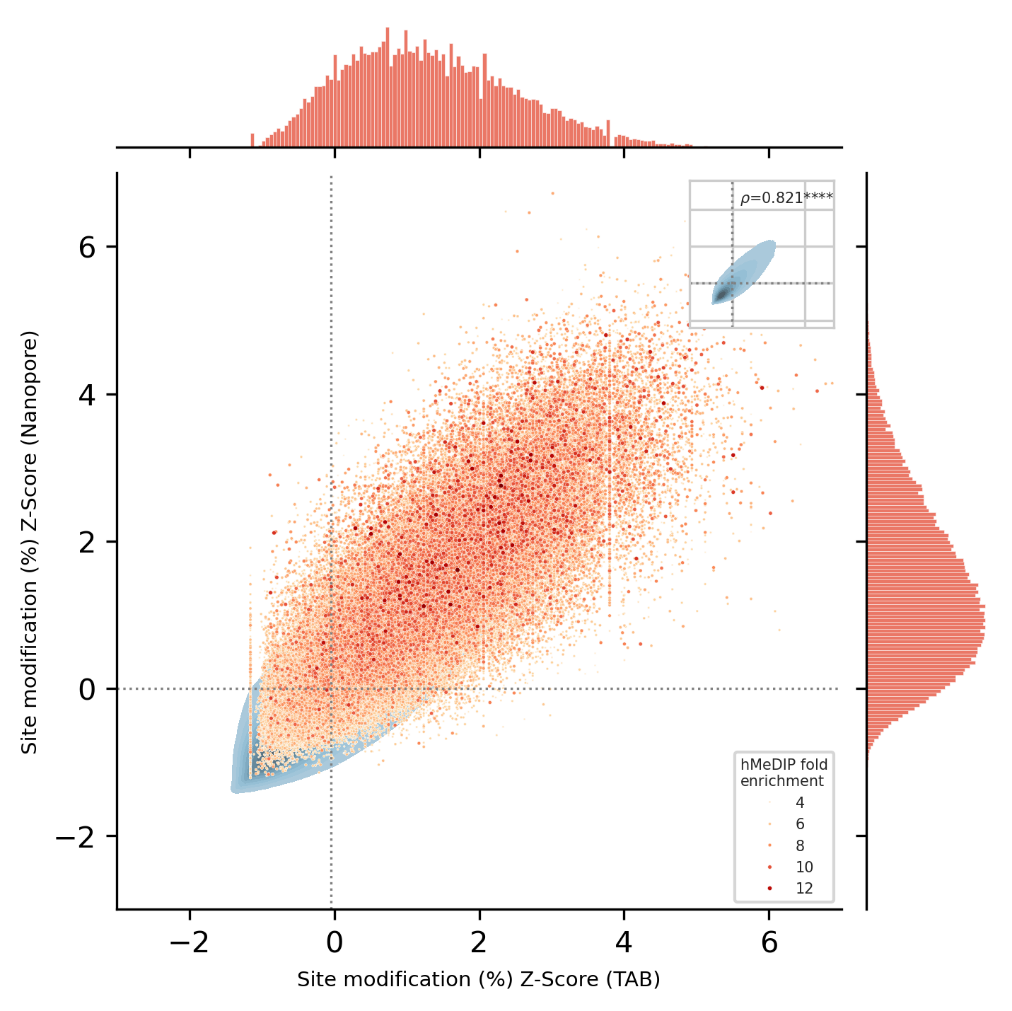


Supplementary Figure 4: Comparison of conventional PCR-based hMeDIP-seq peaks (scatter) and 5hmC detection enrichment from nanopore and TAB-seq (density). CpG-context base-calls from each dataset are aggregated across matched 500bp windows of the genome. The proportion of all base-calls in each window comprised of 5hmC is first arcsine transformed, and then a Z-score is calculated from the transformed values for both nanopore and TAB-seq 5hmC calls. Background plot shows density of those windows, with a scatterplot overlay indicating overlaps with hMeDIP-seq peaks from a public hMeDIP-seq dataset ^1^. Inset plot (top-right) shows background density without scatterplot overlay. Histograms along margins count hMeDIP-seq peaks relative to (top) TAB-seq and (bottom) nanopore sequencing. Dotted line shows mean tile Z-score. **** p < 0.0001


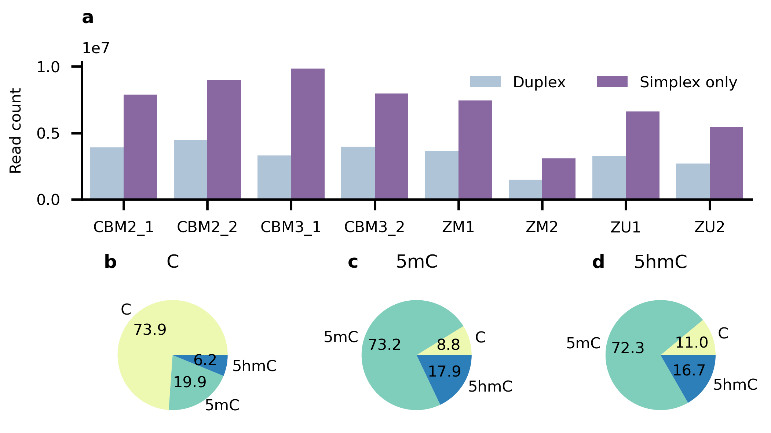


Supplementary Figure 5: Duplex modified base detection in nanopore sequence data. a) Count of all duplex and simplex-only reads across all Nanopore PromethION sequencing runs (N=8), using mouse cerebellum and all methylation standards. b-d) Pie charts show CpG dyad partners from the mu rine duplex datasets (CBM2_1, CBM2_2, CBM3_1, CBM3_2) as a percentage of all CpG dyad pairs containing a) unmodified C, b) 5mC, or c) 5hmC. Duplex base-calls across all replicates are counted.


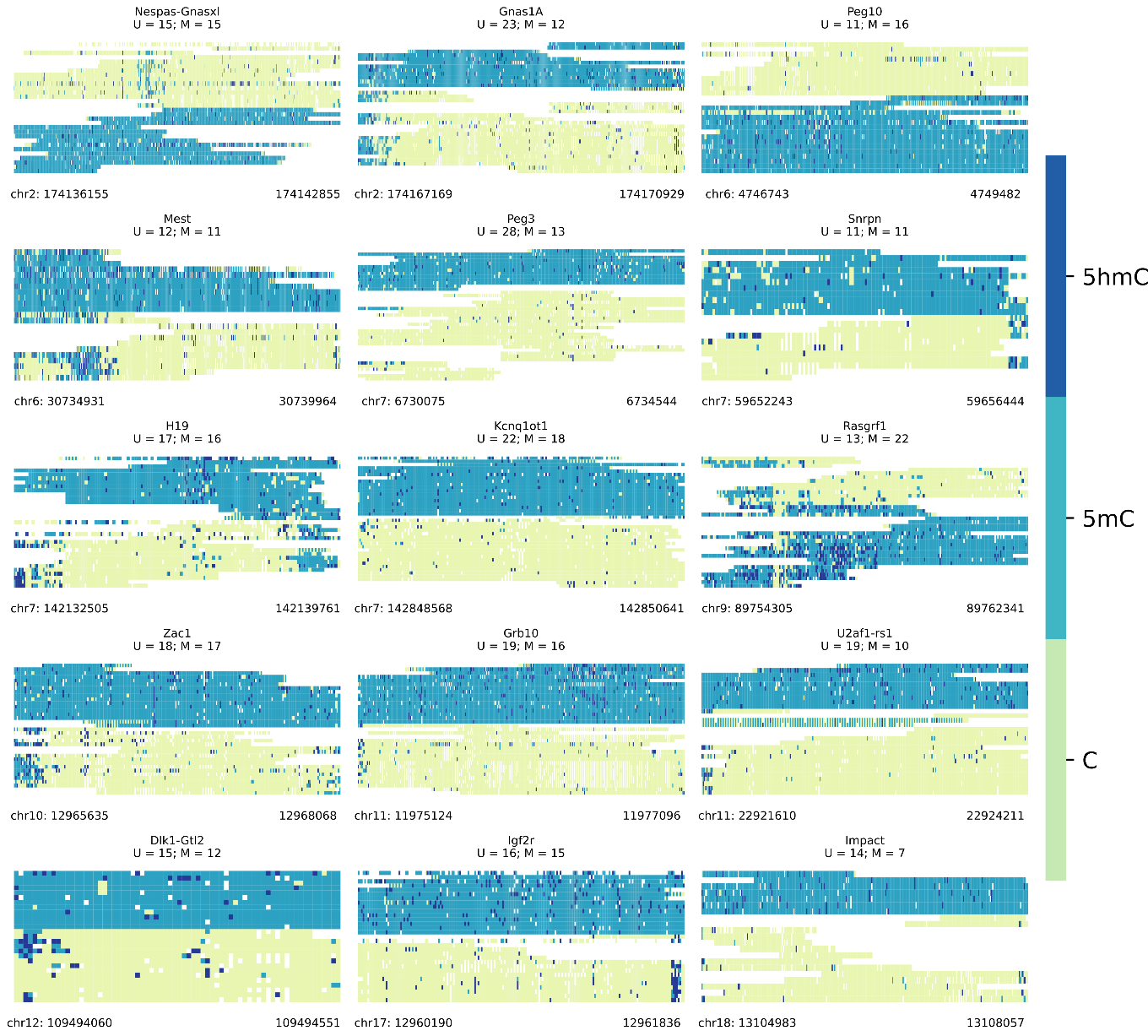


Supplementary Figure 6: Differentially methylated regions from imprinted gene and imprinting clusters in mouse cerebellum, sorted by allele. Duplex reads from across all replicates are concatenated. Heatmap titles contain the official gene symbol, count of reads assigned to the unmethylated allele (U), and count of methylated reads (M).


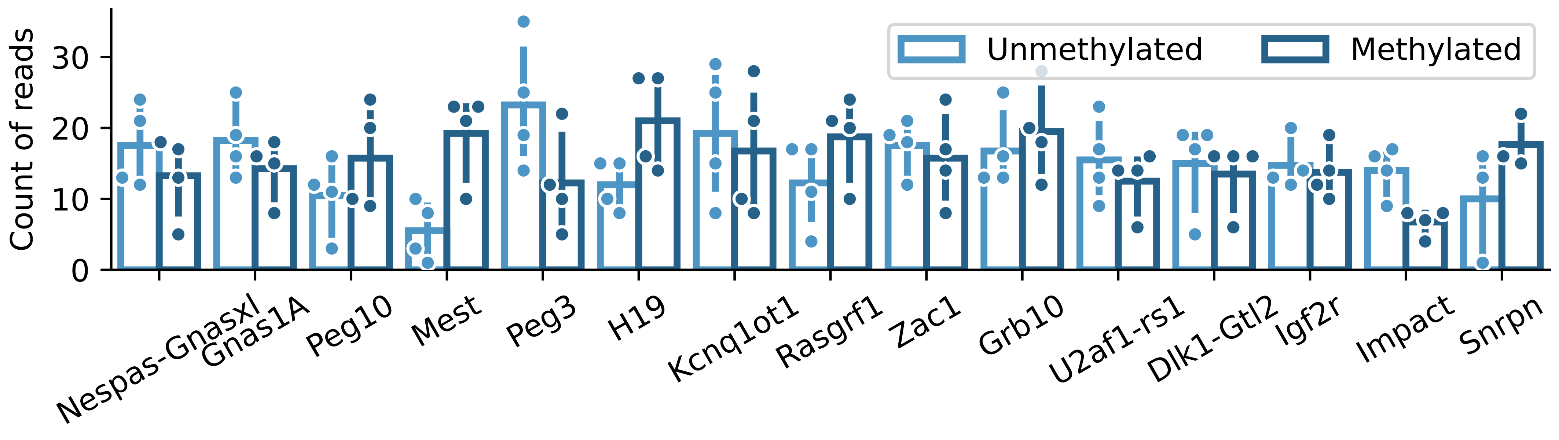


Supplementary Figure 7: Count of reads by allele for imprinted gene and imprinting clusters (N=4).


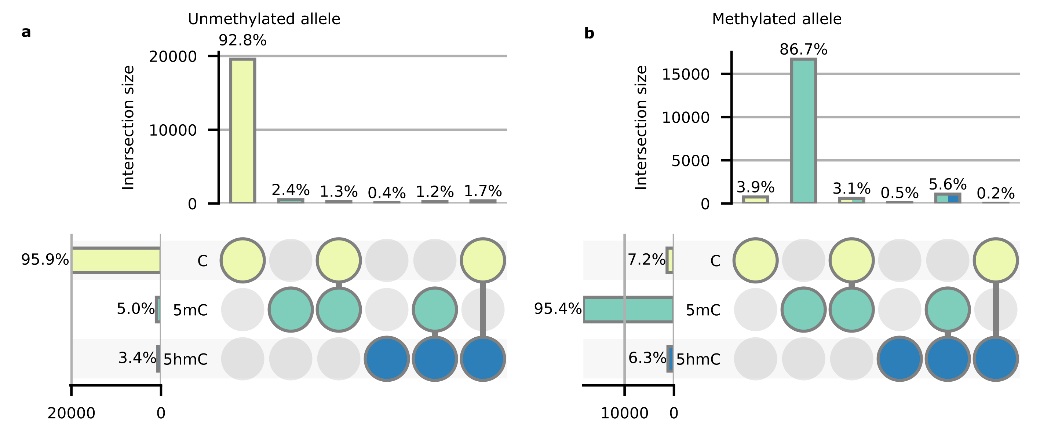


Supplementary Figure 8: Duplex base-calls at the differentially methylated regions of imprinted genes separated by allele. a-b) UpSet plots of duplex modification states on the a) unmethylated and b) methylated allele. Duplex base-calls from all replicates (N=4) are concatenated. Produced using UpSetPlot^2^.

## Supplementary Tables

Supplementary Table 1: Depth and coverage statistics for mouse whole genome sequencing datasets. Genomic depth refers to depth of coverage in a non-strand-specific manner. Depth at CpG is the median depth of individual CpG-context cytosine positions on either strand.

|  | Biological replicate | Technical replicate | Median depth (genomic) | Median depth  (CpG; stranded) |
| --- | --- | --- | --- | --- |
| Nanopore PromethION | CBM2 | (1/2) | 29 | 15 |
|  |  | (2/2) | 33 | 17 |
|  | CBM3 | (1/2) | 32 | 16 |
|  |  | (2/2) | 29 | 16 |
| TAB-seq | 1 | (1/1) | 6 | 3 |
|  | 2 | (1/1) | 8 | 3 |
|  | 3 | (1/1) | 8 | 3 |
| oxBS-seq | 1 | (1/1) | 16 | 6 |
|  | 2 | (1/1) | 5 | 2 |

Supplementary Table 2: Summary statistics for modified base detection in all biological samples.

|  | Biological replicate | Technical replicate | Total CpG base-calls | Percentage detected as modified (%) | |
| --- | --- | --- | --- | --- | --- |
|  |  |  |  | 5mC | 5hmC |
| Nanopore PromethION | CBM2 | (1/2) | 389,659,851 | 62.8 | 9.6 |
|  |  | (2/2) | 442,519,412 | 62.6 | 9.6 |
|  | CBM3 | (1/2) | 446,149,544 | 60.3 | 10.0 |
|  |  | (2/2) | 436,343,532 | 60.5 | 10.4 |
| TAB-seq | (1/3) | (1/1) | 71,651,039 | NA | 11.6 |
|  | (2/3) | (1/1) | 77,410,573 | NA | 11.0 |
|  | (3/3) | (1/1) | 86,804,432 | NA | 11.8 |
| oxBS-seq | (1/2) | (1/1) | 163,400,082 | 68.8 | NA |
|  | (2/2) | (1/1) | 50,089,062 | 66.3 | NA |

Supplementary Table 3: Summary statistics of 5mC and 5hmC base-calls in different genomic contexts.

|  | 5mC | | | | 5hmC | | | |
| --- | --- | --- | --- | --- | --- | --- | --- | --- |
|  | **Median (Z)** | **Mean (%)** | **Median (%)** | **Variance (%)** | **Median (Z)** | **Mean (%)** | **Median (%)** | **Variance (%)** |
| 3UTR | 0.42 | 64.5 | 74.3 | 8.1 | -0.21 | 11.6 | 8.5 | 1.2 |
| 5UTR | -1.49 | 7.8 | 0.0 | 4.5 | -0.88 | 1.8 | 0.0 | 0.4 |
| CGI | -1.77 | 6.7 | 0.0 | 4.1 | -0.86 | 1.6 | 0.0 | 0.3 |
| Exon | 0.11 | 51.5 | 64.4 | 13.8 | -0.49 | 8.7 | 4.9 | 1.2 |
| Genic | 0.08 | 59.2 | 70.5 | 10.5 | -0.22 | 11.7 | 7.7 | 1.5 |
| Intergenic | 0.34 | 68.0 | 77.8 | 7.6 | -0.44 | 8.2 | 5.1 | 0.9 |
| Intron | 0.33 | 61.2 | 71.6 | 9.4 | -0.21 | 12.5 | 8.5 | 1.6 |
| Promoter | -1.49 | 13.0 | 0.0 | 6.8 | -0.88 | 3.3 | 0.0 | 0.6 |
| Sea | 0.36 | 68.4 | 77.3 | 6.9 | -0.27 | 10.5 | 6.8 | 1.3 |
| Shelf | 0.06 | 59.7 | 68.8 | 8.9 | -0.12 | 12.5 | 8.6 | 1.5 |
| Shore | -1.35 | 32.0 | 17.6 | 11.4 | -0.53 | 8.9 | 3.8 | 1.4 |

Supplementary Table 4: Spearman correlation of 5hmC Z-score between matched genomic features in nanopore and TAB-seq data. Z-scores are calculated from the arcsine transformed proportion of all base-calls contained in a feature comrpised of 5hmC.

| ***Feature type*** | ***Spearman correlation ρ*** | ***Number of features compared (n)*** |
| --- | --- | --- |
| **Intron** | 0.863 | 91,415 |
| **Exon** | 0.85 | 28,742 |
| **Promoter** | 0.747 | 8,371 |
| **3UTR** | 0.859 | 8,243 |
| **5UTR** | 0.761 | 877 |
| **Whole gene body** | 0.977 | 19,190 |

Supplementary Table 5: Coordinates used for ICR analysis. Derived from Tomizawa, et al. ^3^. Coordinates were lifted over to mm39 (GRCm39) from mm10 (GRCm38) using UCSC LiftOver.

| *Chromosome* | *Start* | *End* | *ICR Cluster* | *Expressed allele* |
| --- | --- | --- | --- | --- |
| chr2 | 174135847 | 174142870 | Nespas-Gnasxl | M |
| chr2 | 174167880 | 174171044 | Gnas1A | M |
| chr6 | 4745996 | 4749542 | Peg10 | M |
| chr6 | 30733719 | 30740906 | Mest | M |
| chr7 | 6730075 | 6734545 | Peg3 | M |
| chr7 | 59649823 | 59657730 | Snrpn | M |
| chr7 | 142130545 | 142137800 | H19 | P |
| chr7 | 142848568 | 142850642 | Kcnq1ot1 | M |
| chr9 | 89756317 | 89765880 | Rasgrf1 | P |
| chr10 | 12965635 | 12968069 | Zac1 | M |
| chr11 | 11975032 | 11980194 | Grb10 | M |
| chr11 | 22921532 | 22924921 | U2af1-rs1 | M |
| chr12 | 109494035 | 109494599 | Dlk1-Gtl2 | P |
| chr17 | 12959887 | 12962154 | Igf2r | M |
| chr18 | 13104910 | 13108093 | Impact | M |

Supplementary Table 6: Point-Biserial Correlation between CpG dyad modification state and absolute distance from CTCF ChIP-seq peak summits. Positive correlations imply further distance from binding summits, whereas negative correlations imply proximity. CpG dyads greater than 500bp from a peak summit are excluded from calculations.

| ***Dyad pattern*** | ***Point-Biserial Correlation (***$\boldsymbol{r}_{\boldsymbol{pb}}$***)*** | ***Number of dyads compared (n)*** |
| --- | --- | --- |
| **C:C** | -0.32 | 5,167,962 |
| **5mC:5mC** | 0.25 | 1,945,330 |
| **5hmC:5hmC** | 0.04 | 256,189 |
| **C:5mC** | 0.06 | 341,956 |
| **C:5hmC** | 0.01 | 201,858 |
| **5mC:5hmC** | 0.11 | 806,327 |

Works cited

1 Song, C.-X. *et al.* Selective chemical labeling reveals the genome-wide distribution of 5-hydroxymethylcytosine. *Nature Biotechnology* **29**, 68-72 (2011). <https://doi.org:10.1038/nbt.1732>

2 UpSetPlot v. 0.9.0 (2023).

3 Tomizawa, S.-i. *et al.* Dynamic stage-specific changes in imprinted differentially methylated regions during early mammalian development and prevalence of non-CpG methylation in oocytes. *Development* **138**, 811-820 (2011). <https://doi.org:10.1242/dev.061416>
