# Supplementary material for: Double and single stranded detection of 5-methylcytosine and 5-hydroxymethylcytosine with nanopore sequencing
Source: Commun Biol. 2025 Feb 15;8:243. doi: 10.1038/s42003-025-07681-0 (PMC11830040; doi:10.1038/s42003-025-07681-0)
Supplement: Supplementary file 2 — Description of Additional Supplementary Files [file 42003_2025_7681_MOESM2_ESM.pdf]

Description of Additional Supplementary Files

File name: Supplementary Data

Description: Supplementary data for all hypothesis tests performed during analysis. Includes test groups, sample sizes, exact p-values, test statistics, and effect sizes.
