# Supplementary material for: Double and single stranded detection of 5-methylcytosine and 5-hydroxymethylcytosine with nanopore sequencing
Source: Commun Biol. 2025 Feb 15;8:243. doi: 10.1038/s42003-025-07681-0 (PMC11830040; doi:10.1038/s42003-025-07681-0)
Supplement: Supplementary file 3 — Supplementary Data [file 42003_2025_7681_MOESM3_ESM.pdf]

Context	Test type	Test group 1	Test group 2	N (replicates)	n (observations)	Test-statistic	p-Value	DoF	Alternative
5mC false positive rate vs GC%	Pearson correlation				20 (5 percentile bins)	0.855808741	1.97E-12		
5hmC false positive rate vs GC%	Pearson correlation				20 (5 percentile bins)	0.713668642	2.35E-07		
Total False Positive Rate vs. GC%	Pearson correlation				20 (5 percentile bins)	0.763873158	9.80E-09		
Average CpG 5mC%	Welch's T-test	Nanopore (n=4)	oxBS-seq (n=2)	4 (nanopore), 2 (oxBS-seq)		-5.710925845	0.096122054	1	
Average CpG 5hmC%	Welch's T-test	Nanopore (n=4)	TAB-seq (n=3)	4 (nanopore), 3 (TAB-seq)		-4.191036103	0.024023026	3	
Proportion of CpGs with 5hmC% == 0%	Welch's T-test	Nanopore (n=4)	TAB-seq (n=3)	4 (nanopore), 3 (TAB-seq)		-4.834351673	0.009951938	4	
CpG 5mC% correlation	Spearman correlation	Nanopore (replicates merged)	oxBS-seq (replicates merged)		15,385,941	0.726809839	0	-	
CpG 5hmC% correlation	Spearman correlation	Nanopore (replicates merged)	TAB-seq (replicates merged)		11,241,562	0.50372012	0	-	
Feature-wise comparison: Whole genes	Spearman correlation	Whole genes (nanopore replicates merged)	Whole genes (TAB-seq replicates merged)		19,190	0.877017145	0	-	
Feature-wise comparison: Promoters	Spearman correlation	Promoter (nanopore replicates merged)	Promoter (TAB-seq replicates merged)		8,371	0.747086871	0	-	
Feature-wise comparison: 5UTRs	Spearman correlation	5UTR (nanopore replicates merged)	5UTR (TAB-seq replicates merged)		877	0.761158926	1.02E-166	-	
Feature-wise comparison: Introns	Spearman correlation	Intron (nanopore replicates merged)	Intron (TAB-seq replicates merged		91,415	0.862688981	0	-	
Feature-wise comparison: Exons	Spearman correlation	Exon (nanopore replicates merged)	Exon (TAB-seq replicates merged)		28,742	0.849704339	0	-	
Feature-wise comparison: 3UTRs	Spearman correlation	3UTR (nanopore replicates merged)	3UTR (TAB-seq replicates merged)		8,243	0.859010433	0	-	
Correlation nanopore 5hmC enrichment and hMeDIP fold enrichment	Spearman correlation	Genomic windows from nanopore WGS) (4 replicates merged)	Public hMeDIP-seq peaks (3 replicates merged) fold enrichment		274,295	0.16	0		
Expected vs. observed genomic context: Intergenic	Binomial	Genome average (replicates merged	Nanopore direct hMeDIP-seq peaks		17,368	0.19317135	0		Less
Expected vs. observed genomic context: Intron	Binomial	Genome average (replicates merged	Nanopore direct hMeDIP-seq peaks		17,368	0.641524643	0		Greater
Expected vs. observed genomic	Binomial	Genome average (replicates merged	Nanopore direct hMeDIP-seq peaks		17,368	0.146533855	0		Greater
Expected vs. observed genomic context: Promoter	Binomial	Genome average (replicates merged	Nanopore direct hMeDIP-seq peaks		17,368	0.018770152	3.80E-21		Greater
Proportion of base-calls in WGS vs hMeDIP-seq: C	Welch's T-test	Nanopore WGS (n=4).	Nanopore direct hMeDIP-seq peaks	4 (nanopore WGS), 3 (hMeDIP-seq)		2.356980606	0.066720874	5	
Proportion of base-calls in WGS vs hMeDIP-seq: 5mC	Welch's T-test	Nanopore WGS (n=4)	Nanopore direct hMeDIP-seq (n=3)	4 (nanopore WGS), 3 (hMeDIP-seq)		8.583503538	0.00533192	3	
Proportion of base-calls in WGS vs hMeDIP-seq: 5hmC	Welch's T-test	Nanopore WGS (n=4)	Nanopore direct hMeDIP-seq (n=3)	4 (nanopore WGS), 3 (hMeDIP-seq)		-12.42784378	0.005482809	2	
Independence in observed frequencies of CpG dyad state between DMRs and genome average	G-test	Genome average (replicates merged) (n=4)	Differentially modified regions from genome average (replicates merged)		-	1030.444353	4.00E-217	8	
Dyad state as proportion of base-calls: C:C	Paired T-test	Genome average (replicates merged) (n=4)	CTCF motifs (all) (n=4)		4	11.75199258	0.001324128	3	
Dyad state as proportion of base-calls: 5mC:5mC	Paired T-test	Genome average (replicates merged) (n=4)	CTCF motifs (all) (n=4)		4	-9.006379564	0.002889839	3	
Dyad state as proportion of base-calls: 5hmC:5hmC	Paired T-test	Genome average (replicates merged) (n=4)	CTCF motifs (all) (n=4)		4	-2.297500364	0.10522933	3	
Dyad state as proportion of base-calls: C:5mC	Paired T-test	Genome average (replicates merged) (n=4)	CTCF motifs (all) (n=4)		4	-15.31206045	0.00060498	3	
Dyad state as proportion of base-calls: C:5hmC	Paired T-test	Genome average (replicates merged) (n=4)	CTCF motifs (all) (n=4)		4	-0.268256716	0.805890007	3	

Dyad state as proportion of base-calls: 5mC:5hmC	Paired T-test	Genome average (replicates merged) (n=4)	CTCF motifs (all) (n=4)		4	-16.02405153	0.000528565	3	
Dyad state as proportion of base-calls: C:C	Paired T-test	Genome average (replicates merged) (n=4)	CTCF motifs (at ChIP summits) (n=4)		4	93.81556233	2.67E-06	3	
Dyad state as proportion of base-calls: 5mC:5mC	Paired T-test	Genome average (replicates merged) (n=4)	CTCF motifs (at ChIP summits) (n=4)		4	53.35232565	1.45E-05	3	
Dyad state as proportion of base-calls: 5hmC:5hmC	Paired T-test	Genome average (replicates merged) (n=4)	CTCF motifs (at ChIP summits) (n=4)		4	-28.40167662	9.58E-05	3	
Dyad state as proportion of base-calls: C:5mC	Paired T-test	Genome average (replicates merged) (n=4)	CTCF motifs (at ChIP summits) (n=4)		4	-35.71627455	4.83E-05	3	
Dyad state as proportion of base-calls: C:5hmC	Paired T-test	Genome average (replicates merged) (n=4)	CTCF motifs (at ChIP summits) (n=4)		4	-11.52703956	0.001401762	3	
Dyad state as proportion of base-calls: 5mC:5hmC	Paired T-test	Genome average (replicates merged) (n=4)	CTCF motifs (at ChIP summits) (n=4)		4	-97.06784652	2.41E-06	3	
Dyad state as proportion of base-calls: C:C	Paired T-test	CTCF motifs (all) (n=4)	CTCF motifs (at ChIP summits) (n=4)		4	64.84490809	8.08E-06	3	
Dyad state as proportion of base-calls: 5mC:5mC	Paired T-test	CTCF motifs (all) (n=4)	CTCF motifs (at ChIP summits) (n=4)		4	-47.04032326	2.12E-05	3	
Dyad state as proportion of base-calls: 5hmC:5hmC	Paired T-test	CTCF motifs (all) (n=4)	CTCF motifs (at ChIP summits) (n=4)		4	-38.67925885	3.80E-05	3	
Dyad state as proportion of base-calls: C:5mC	Paired T-test	CTCF motifs (all) (n=4)	CTCF motifs (at ChIP summits) (n=4)		4	-29.90366737	8.21E-05	3	
Dyad state as proportion of base-calls: C:5hmC	Paired T-test	CTCF motifs (all) (n=4)	CTCF motifs (at ChIP summits) (n=4)		4	-27.1266351	0.000109942	3	
Dyad state as proportion of base-calls: 5mC:5hmC	Paired T-test	CTCF motifs (all) (n=4)	CTCF motifs (at ChIP summits) (n=4)		4	-173.4968282	4.22E-07	3	
Dyad state difference in absolute distance from ChIP summit	Kruskal-Wallace	C:C, 5mC:5mC, 5hmC:5hmC, C:5mC, C:5hmC, 5mC:5hmC	-			953630.1071	0.00E+00	-	
Post hoc Dunn analysis of Kruskal-Wallace	Dunn test	C:C, 5mC:5mC, 5hmC:5hmC, C:5mC, C:5hmC, 5mC:5hmC	-			-	-	-	

Dunn post hoc analysis	5hmC:5hmC	5mC:5hmC	5mC:5mC	C:5hmC	C:5mC	C:C
**5hmC:5hmC**	1.00E+00	0.00E+00	0.00E+00	0.00E+00	1.12E-90	0.00E+00
**5mC:5hmC**	0.00E+00	1.00E+00	0.00E+00	0.00E+00	2.16E-201	0.00E+00
**5mC:5mC**	0.00E+00	0.00E+00	1.00E+00	0.00E+00	0.00E+00	0.00E+00
**C:5hmC**	0.00E+00	0.00E+00	0.00E+00	1.00E+00	0.00E+00	0.00E+00
**C:5mC**	1.12E-90	2.16E-201	0.00E+00	0.00E+00	1.00E+00	0.00E+00
**C:C**	0.00E+00	0.00E+00	0.00E+00	0.00E+00	0.00E+00	1.00E+00
