# Supplementary material for: Double and single stranded detection of 5-methylcytosine and 5-hydroxymethylcytosine with nanopore sequencing
Source: Commun Biol. 2025 Feb 15;8:243. doi: 10.1038/s42003-025-07681-0 (PMC11830040; doi:10.1038/s42003-025-07681-0)
Supplement: Supplementary file 4 — Reporting Summary [file 42003_2025_7681_MOESM4_ESM.pdf]

nature portfolio

Corresponding author(s): Dominic Oliver Halliwell

Last updated by author(s): Jan 30, 2025

Reporting Summary

Nature Portfolio wishes to improve the reproducibility of the work that we publish. This form provides structure for consistency and transparency in reporting. For further information on Nature Portfolio policies, see our Editorial Policies and the Editorial Policy Checklist.

Statistics

For all statistical analyses, confirm that the following items are present in the figure legend, table legend, main text, or Methods section.

n/a	Confirmed	
☐	☒	The exact sample size (*n*) for each experimental group/condition, given as a discrete number and unit of measurement
☐	☒	A statement on whether measurements were taken from distinct samples or whether the same sample was measured repeatedly
☐	☒	The statistical test(s) used AND whether they are one- or two-sided<br>*Only common tests should be described solely by name; describe more complex techniques in the Methods section.*
☒	☐	A description of all covariates tested
☐	☒	A description of any assumptions or corrections, such as tests of normality and adjustment for multiple comparisons
☐	☒	A full description of the statistical parameters including central tendency (e.g. means) or other basic estimates (e.g. regression coefficient) AND variation (e.g. standard deviation) or associated estimates of uncertainty (e.g. confidence intervals)
☐	☒	For null hypothesis testing, the test statistic (e.g. *F*, *t*, *r*) with confidence intervals, effect sizes, degrees of freedom and *P* value noted<br>*Give P values as exact values whenever suitable.*
☒	☐	For Bayesian analysis, information on the choice of priors and Markov chain Monte Carlo settings
☒	☐	For hierarchical and complex designs, identification of the appropriate level for tests and full reporting of outcomes
☐	☒	Estimates of effect sizes (e.g. Cohen's *d*, Pearson's *r*), indicating how they were calculated

Our web collection on statistics for biologists contains articles on many of the points above.

Software and code

Policy information about availability of computer code

Data collection	Machine data was collected using Oxford Nanopore Technologies MinKNOW software (v23.11.4). This was base-called using Oxford Nanopore Technologies Dorado basecaller (v0.5.1). Modified bases were extracted using Oxford Nanopore Technologies Modkit (v0.2.8).
Data analysis	A variety of open source and custom code was used to analyse data. This includes samtools (v1.19) and bedtools (v2.31.1). Python (v3.9.18) packages are included by name and version in the manuscript, and are also visible on the published project GitHub repository as an environment.yaml file. These include pandas (2.0.1), numpy (1.24.3), scipy (1.13.1), scikit-learn (1.5.2), PyRanges (0.0.120), and Pingouin (0.5.4). Visualisations were produced using matplotlib (3.7.1), seaborn (0.13.2), pyGenomeTracks (3.8), and upsetplot (v0.9.0). The Integrative Genomics Viewer (IGV)(2.19.1) is also used extensively.<br><br>MACS2 (v.2.2.6) is used for peak detection. Trimming is performed using TrimGalore! (v0.6.10).<br><br>Custom code is published in the main project GitHub repository (v1.0.0): https://doi.org/10.5281/zenodo.14753744. Other custom code includes CHIP2MACS2 (v1.0.0) (https://doi.org/10.5281/zenodo.14535832), which was made as a standalone pipeline for processing ChIP and DIP-seq. data

For manuscripts utilizing custom algorithms or software that are central to the research but not yet described in published literature, software must be made available to editors and reviewers. We strongly encourage code deposition in a community repository (e.g. GitHub). See the Nature Portfolio guidelines for submitting code & software for further information.

Data

Policy information about availability of data

All manuscripts must include a data availability statement. This statement should provide the following information, where applicable:

- Accession codes, unique identifiers, or web links for publicly available datasets
- A description of any restrictions on data availability
- For clinical datasets or third party data, please ensure that the statement adheres to our policy

Raw nanopore machine data in fast5 format has been made available for all murine whole genome sequence experiments on the Sequence Read Archive (SRA) as BioProject PRJNA1144670. Aligned sequence data in BAM file format is also available. Additionally, CpG context modified base detections, as produced by 'modkit pileup' are available on the NCBI GEO archive with the accession: GSE279860. These data are limited to CpG positions relative to the mm39 reference genome and are soft-masked. Machine data is not available for the Zymo DNA Methylation Standards; however, these are available as BAM format files under the same BioProject as above.

For the nanopore hMeDIP-seq experiments, data is available in both BAM format under the previously mentioned SRA BioProject, as well as in pod5 format on Zenodo, with record DOI: 10.5281/zenodo.14514705. BAM format sequence data used as an input is available as SRR30150148 on the SRA.
Source data for figures can be downloaded from Figshare (DOI: 10.6084/m9.figshare.28287962).

Research involving human participants, their data, or biological material

Policy information about studies with human participants or human data. See also policy information about sex, gender (identity/presentation), and sexual orientation and race, ethnicity and racism.

Reporting on sex and gender	No human participants or human data is used in this study.<br><br>Human biological material is limited to the commercially available Zymo DNA Methylation Standard samples, for which no information on sex or gender is relevant or reported in this study.
Reporting on race, ethnicity, or other socially relevant groupings	NA
Population characteristics	NA
Recruitment	NA
Ethics oversight	NA

Note that full information on the approval of the study protocol must also be provided in the manuscript.

Field-specific reporting

Please select the one below that is the best fit for your research. If you are not sure, read the appropriate sections before making your selection.

☒ Life sciences ☐ Behavioural & social sciences ☐ Ecological, evolutionary & environmental sciences

For a reference copy of the document with all sections, see nature.com/documents/nr-reporting-summary-flat.pdf

Life sciences study design

All studies must disclose on these points even when the disclosure is negative.

Sample size	No statistical calculation was used to predetermine sample size. For the commercially available DNA methylation standards, sequencing was performed in duplicate on both the unmodified and methylated WGA standards. For ex vivo mouse tissues, Nanopore sequence data was produced in two biological replicates, sequenced each in duplicate (total N=4). We planned for considerable sequencing depth using a Nanopore PromethION sequencing platform, and considered that at this depth, this sample size would be sufficient to detect large-scale differences in modification detection between sequencing methods, as well as between individual biological and technical replicates.
Data exclusions	Data exclusion was performed on sequencing depth. Genomic positions with a sequencing depth below a predefined threshold of 5x (strand specific) were excluded from comparison. This was determined as low sequencing depth positions had a tendency for introducing outliers to the data, and higher depth positions were considered to provide a more reliable representation of the underlying modification state at a position.
Replication	Intra-assay variation was measured between Nanopore, TAB-seq, and oxBS-seq replicates at CpG site level using Root Mean Square Deviation (RMSD). This was calculated pairwise (replicate-by-replicate), before the mean of these statistics was produced to provide a summary.

Randomization	This is not relevant to this study. No group allocation process exists. All samples are in identical condition (age, sex, health, mouse strain, etc.) and are different only in the technique used for DNA sequencing.
Blinding	Blinding is not relevant to this study. No investigators exist nor any kind of group allocation system.

Reporting for specific materials, systems and methods

We require information from authors about some types of materials, experimental systems and methods used in many studies. Here, indicate whether each material, system or method listed is relevant to your study. If you are not sure if a list item applies to your research, read the appropriate section before selecting a response.

Materials & experimental systems

n/a	Involved in the study	
☐	☒	Antibodies
☒	☐	Eukaryotic cell lines
☒	☐	Palaeontology and archaeology
☐	☒	Animals and other organisms
☒	☐	Clinical data
☒	☐	Dual use research of concern
☒	☐	Plants

Methods

n/a	Involved in the study	
☐	☒	ChIP-seq
☒	☐	Flow cytometry
☒	☐	MRI-based neuroimaging

Antibodies

Antibodies used	Whole serum α-5hmC antibody (ActiveMotif, 39769; lot 23720003). 1 µL (1:500) per reaction.<br>IgG-purified α-5hmC antibody (ActiveMotif, 39791; lot 25419010; concentration: 1 µg/µL). 2 µL (1:250) per reaction.
Validation	Active Motif have validated the use of both antibodies for different species, including mouse (listed as "Not Species Specific" on manufacturer website). Active Motif has used qPCR and dot-blot assays to confirm specific recognition of 5hmC. These antibodies are recommended in the widely used hMeDIP-seq protocol described in Nestor, C.E. and Meehan, R.R., 2014. Hydroxymethylated DNA Immunoprecipitation (hmeDIP). In: J.C. Stockert, J. Espada and A. Blázquez-Castro, eds. Functional Analysis of DNA and Chromatin. Totowa, NJ: Humana Press, pp. 259-267. These have been used in at least 75 published papers showcasing hMeDIP-seq according to the manufacturer.<br><br>Per the manufacturer's website: "Active Motif offers two polyclonal antibodies that recognize 5-hydroxymethylcytosine, a whole serum version (39769) and a purified IgG version (39791).Both are validated for use in methyl DNA immunoprecipitation (MeDIP). For customers that require the ability to quantitate the amount of IgG in the MeDIP reaction, the purified IgG version (39791) is recommended. The whole serum version (39769) is very high titre and should be used carefully (0.1 - 0.5 µl per IP) as not to generate high non-specific background. The whole serum version (39769) has been used successfully in immunofluorescence (IF, Ito et al, 2010), and the purified IgG version (39791) is likely to work in this application as well."

Animals and other research organisms

Policy information about studies involving animals; ARRIVE guidelines recommended for reporting animal research, and Sex and Gender in Research

Laboratory animals	The study involved tissues from three mice: Mus musculus, C57Bl/6Cr (Charles River Laboratories), 8 weeks from birth.
Wild animals	The study did not involve wild animals.
Reporting on sex	Findings apply to one sex only. In study design, it was determined that tissue from a single sex would be used to reduce epigenetic heterogeneity between samples, which could be mistaken for error. Furthermore, we selected an all-female cohort, in order to match the cohort used in a publicly available dataset (oxBS-seq and TAB-seq in Ma, Q., Lu, H., Xu, Z., Zhou, Y. and Ci, W., 2017. Mouse olfactory bulb methylome and hydroxymethylome maps reveal noncanonical active turnover of DNA methylation. Epigenetics, 12(8), pp. 708-714). By having samples homogenous to those used in that study, we could more reliably compare datasets produced using different techniques with less influence from sex specific differences in epigenetic modification.
Field-collected samples	The study did not involve samples collected from the field.
Ethics oversight	The use of research animals for this research was given a favourable opinion by the University of Bath's Animal Welfare and Ethical Review Body (AWERB; Review Reference 3436-4022), a Committee defined in law under the UK Animals (Scientific Procedures) Act, and approved by the University's Academic Ethics and Integrity Committee (AEIC).

Note that full information on the approval of the study protocol must also be provided in the manuscript.

Plants

Seed stocks

Report on the source of all seed stocks or other plant material used. If applicable, state the seed stock centre and catalogue number. If plant specimens were collected from the field, describe the collection location, date and sampling procedures.

Novel plant genotypes

Describe the methods by which all novel plant genotypes were produced. This includes those generated by transgenic approaches, gene editing, chemical/radiation-based mutagenesis and hybridization. For transgenic lines, describe the transformation method, the number of independent lines analyzed and the generation upon which experiments were performed. For gene-edited lines, describe the editor used, the endogenous sequence targeted for editing, the targeting guide RNA sequence (if applicable) and how the editor was applied.

Authentication

Describe any authentication procedures for each seed stock used or novel genotype generated. Describe any experiments used to assess the effect of a mutation and, where applicable, how potential secondary effects (e.g. second site T-DNA insertions, mosiacism, off-target gene editing) were examined.

ChIP-seq

Data deposition

☒ Confirm that both raw and final processed data have been deposited in a public database such as GEO.

☒ Confirm that you have deposited or provided access to graph files (e.g. BED files) for the called peaks.

Data access links
May remain private before publication.

BAM format sequence data of the treatment and input files are available on the NCBI SRA in PRJNA1144670, under BioSample: SAMN43041789. Pod5 format machine data is available for all treatment experiments on Zenodo: https://doi.org/10.5281/zenodo.14514704.

Narrow peak files are available for each experiment on Figshare (as part of Figure 3 source data): doi.org/10.6084/m9.figshare.28287962.v2 (downloadable in fig3.tar.gz) AND on the GEO archive under GEO Series GSE288331, where direct modified base detections are also available for these peaks in bed format.

Files in database submission

SRA:
s3://sra-pub-src-4/SRR30150151/cbm1_ip_13_07_23_basecalls.bam
s3://sra-pub-src-4/SRR30150150/cbm1_ip_rep1_23_16_08.bam
s3://sra-pub-src-8/SRR30150149/cbm1_ip_rep2_23_05_09.bam
s3://sra-pub-src-3/SRR30150148/cbm1_input.bam

Figshare:
fig3a_pygenometracks.tar/hmedip_rep0_peaks.narrowPeak
fig3a_pygenometracks.tar/hmedip_rep1_peaks.narrowPeak
fig3a_pygenometracks.tar/hmedip_rep2_peaks.narrowPeak

GEO:
hmedip_rep1_peaks.narrowPeak.gz
hmedip_rep2_peaks.narrowPeak.gz
hmedip_rep3_peaks.narrowPeak.gz
rep1_peak_pileup.bed.gz
rep2_peak_pileup.bed.gz
rep3_peak_pileup.bed.gz

Note: due to a naming issue: hmedip_rep0_peaks.narrowPeak and hmedip_rep1_peaks.narrowPeak are actually the same file.

Zenodo (rep. 3 divided into 5 files due to individual file size constraints):
CBM1_hMeDIP1.tar.gz
CBM1_hMeDIP2.tar.gz
CBM1_hMeDIP3.01.tar
CBM1_hMeDIP3.02.tar
CBM1_hMeDIP3.03.tar
CBM1_hMeDIP3.04.tar
CBM1_hMeDIP3.05.tar

Genome browser session
(e.g. UCSC)

No longer applicable.

Methodology

Replicates

Three experimental replicates are used (treatment). One control replicate was sequenced as an input.

Sequencing depth

Sequencing was performed using an Oxford Nanopore Technologies MinION sequencer (effectively single-end). Each replicate used a single MinION flow cell. Reads were first sheared to 10kb, and then sonicated to a mean fragment length of 362 bp.

For replicates 1-3: total reads: 977089, 1753849, 4159865; of which, mapped: 802584, 599414, 465264.

Antibodies	Whole serum α-5hmC antibody (ActiveMotif, 39769)<br>IgG-purified α-5hmC antibody (ActiveMotif, 39791)
Peak calling parameters	MACS2 (v2.2.6) with default settings:<br><br>macs2 callpeak <treatment bam> <input bam> --gsize mm
Data quality	macs2 callpeak default settings include FDR threshold of q<0.05 (where q = p-adjusted with Benjamini-Hochberg multiple hypothesis testing).
Software	MACS2 (v2.2.6) to call peaks. Custom code includes CHIP2MACS2 (v1.0.0) (https://doi.org/10.5281/zenodo.14535832), a pipeline that includes read trimming, sorting, alignment, and removal of blacklisted segments. Duplicates are marked using Picard
